# Tetrazine Amino Acid Encoding for Rapid and Complete Protein Bioconjugation

**DOI:** 10.21769/BioProtoc.5048

**Published:** 2024-08-20

**Authors:** Alex J. Eddins, Abigail H. Pung, Richard B. Cooley, Ryan A. Mehl

**Affiliations:** 1 Department of Biochemistry and Biophysics, Oregon State University, 2011 Agricultural and Life Sciences, Corvallis, OR, USA; 2 GCE4All Research Center, Oregon State University, 2011 Agricultural and Life Sciences, Corvallis, OR, USA

**Keywords:** 1, 2, 4, 5-tetrazine (Tet), Noncanonical amino acid (ncAA), Genetic code expansion (GCE), Bioorthogonal, Near-cognate suppression, Strained *trans*-cyclooctene (sTCO), Aminoacyl tRNA synthetase/tRNA pair (aaRS/tRNA)

## Abstract

Generating protein conjugates using the bioorthogonal ligation between tetrazines and *trans*-cyclooctene groups avoids the need to manipulate cysteine amino acids; this ligation is rapid, site-specific, and stoichiometric and allows for labeling of proteins in complex biological environments. Here, we provide a protocol for the expression of conjugation-ready proteins at high yields in *Escherichia coli* with greater than 95% encoding and labeling fidelity. This protocol focuses on installing the Tet2 tetrazine amino acid using an optimized genetic code expansion (GCE) machinery system, Tet2 pAJE-E7, to direct Tet2 encoding at TAG stop codons in BL21 *E. coli* strains, enabling reproducible expression of Tet2-proteins that quantitatively react with trans-cyclooctene (TCO) groups within 5 min at room temperature and physiological pH. The use of the BL21 derivative B95(DE3) minimizes premature truncation byproducts caused by incomplete suppression of TAG stop codons, which makes it possible to use more diverse protein construct designs. Here, using a superfolder green fluorescent protein construct as an example protein, we describe in detail a four-day process for encoding Tet2 with yields of ~200 mg per liter of culture. Additionally, a simple and fast diagnostic gel electrophoretic mobility shift assay is described to confirm Tet2-Et encoding and reactivity. Finally, strategies are discussed to adapt the protocol to alternative proteins of interest and optimize expression yields and reactivity for that protein.

Key features

• Protocol describes site-specific encoding of the tetrazine amino acid Tet2-Et into proteins for bioorthogonal, quantitative, and rapid attachment of *trans*-cyclooctene-containing labels.

• Protocol uses auto-induction methods for the production Tet2-Et protein in *E. coli.*

• This protocol focuses on Tet-protein expressions in BL21(DE3) and B95(DE3) strains, which take approximately 4 days to complete.

• SDS-PAGE mobility shift assay using a strained TCO-PEG_5000_ (sTCO-PEG_5000_) reagent provides a simple, generalizable method for testing Tet-protein reactivity.

## Graphical overview


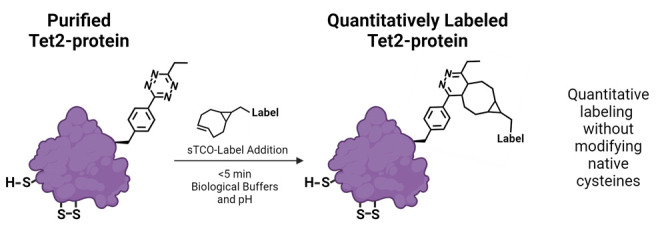


## Background

The facile ability to quantitatively and rapidly attach molecules onto proteins without needing to remove or modify native cysteine residues would greatly advance the study of proteins and the development of homogeneous protein reagents, therapeutics, and diagnostics. Previously, we have demonstrated that site-specific, translational encoding of tetrazine noncanonical amino acids (Tet-ncAAs) into proteins via genetic code expansion (GCE) provides the required qualities of ideal bioorthogonal reactions to advance protein labeling [1,2]. Three of these essential qualities are (1) an exceptionally fast bioorthogonal reaction that occurs under biological conditions enabling complete labeling in short reaction times, (2) a high-fidelity GCE system that ensures that all encoding sites contain the ncAA, and (3) the ncAA is stable during encoding in biological environments so that all sites are reactive. While a variety of encodable labeling strategies are available [3,4], the encoding of Tet-ncAAs into proteins and subsequent attachment of labels containing a *trans*-cyclooctene (TCO) functional group stands out as the only strategy that has these three qualities for routine, quantitative protein labeling. These advantages have been leveraged to produce systems for homogeneous protein conjugation to surfaces [5,6], highly effective anti-viral nanobody conjugates [2], and the attachment of spectroscopic probes both in vitro [7] and in vivo [8].

**Figure 1.**
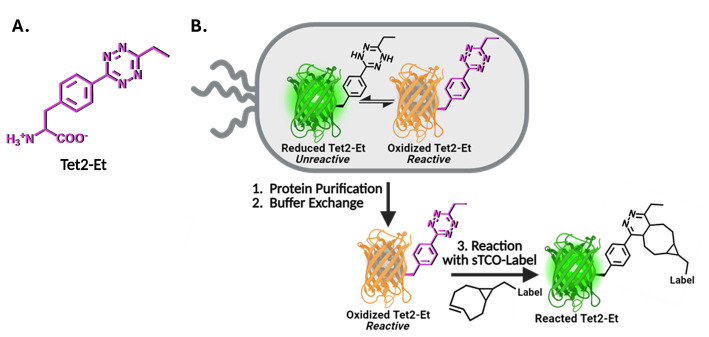
Genetic code expansion (GCE) encoding of tetrazine noncanonical amino acids (Tet-ncAA) and quantitative reaction with *trans*-cyclooctene (TCO) labels. (A) Structure of the Tet2-Et ncAA in its oxidized, reactive state. (B) During protein expression, sfGFP^150^ with encoded Tet2-Et exists in an equilibrium between an unreactive reduced state (green, top left) and the reactive oxidized state (orange, top right). The orange color of reactive sfGFP^Tet2-Et ^results from Tet2 quenching of sfGFP fluorescence when Tet2-Et is encoded at site N150. This fluorescence quenching does not occur when Tet2-Et is in the reduced form. Upon purification, buffer exchange, and exposure to ambient oxygen, any reduced (green) Tet2-sfGFP protein quickly oxidizes and can be quantitatively reacted with the desired sTCO labeling reagent. After reaction with sTCO, Tet2-Et no longer quenches sfGFP fluorescence. See General notes 1 and 2 for more information on Tet2 reactions and redox properties, respectively.

Here, we describe the GCE encoding of an ethyl-substituted 1,2,4,5-tetrazine ncAA (Tet2-Et) and its in vitro reaction with sTCO labels having a second-order rate constant of ~10^4^ M^-1^·s^-1^ at room temperature and physiological pH (Figure 1). We use an engineered Tet2-Et RS/tRNA pair with optimized efficiency and fidelity and plasmid construct that provides high-fidelity encoding (i.e., > 95% encoding accuracy) and quantitative labeling [1]. This protocol outlines the expression of a control protein, superfolder green fluorescent protein (sfGFP) with a TAG codon at the N150 site (sfGFP^150^ [9]) that yields Tet-containing sfGFP (sfGFP^Tet2-Et^) with yields of ~200 mg per liter of culture (Figure 1, Table 1). This Tet2-Et expression system requires two plasmids: (1) the pAJE-E7 GCE machinery plasmid expressing the Tet2-Et RS/tRNA pair, and (2) a plasmid that expresses a gene of interest containing the TAG stop codon at the intended site of Tet encoding. Compatible expression hosts include the standard IPTG/lactose-inducible BL21(DE3) cell line or B95(DE3) *ΔAΔfabR*, which minimizes premature protein truncation at TAG-encoding sites [10]. Both strains are compatible with target protein expression from classical pET vectors (e.g., pET28). Expressions are performed in auto-induction media (AIM) for reproducible, high-yielding Tet2-protein production. Finally, we describe an electrophoresis mobility shift assay using TCO-functionalized PEG_5000_ for quick confirmation of accurate Tet encoding into proteins, the stability of TCO reagents, and the efficiency of protein conjugation. Completion of this protocol, including the confirmation of Tet2-Et encoding into sfGFP, takes approximately four days. Discussions on adapting this protocol for encoding Tet2-Et into biologically relevant proteins are provided.

## Materials and reagents

**Biological materials**

**Strains**

1. BL21(DE3) (Thermo Fisher, catalog number: EC0114). This strain of *E. coli* is optimized for over-expression of target proteins under the T7 transcriptional promoter that is commonly found in standard pET vectors. This strain contains a genomic copy of the T7 RNA polymerase controlled by the lacUV5 promoter so that upon introduction with IPTG, the T7 polymerase is expressed and transcribes the target gene to produce high quantities of recombinant target protein. This strain contains Release Factor 1 (RF1), the protein responsible for terminating translation at TAG amber stop codons so that when encoding Tet2-Et at TAG codons of target proteins, truncated protein will be produced along with full-length Tet2-protein. To avoid co-purification of truncated protein with full-length protein, C-terminal purification tags are recommended. For proteins that self-assemble into homo-multimers (dimers, trimers, etc.), purification can be challenging due to the possible co-purification of truncated forms that are incorporated as subunits in the assembly. If using N-terminal purification/solubilization tags, additional purification steps may be needed to remove truncated protein species. See Troubleshooting #1 for more discussion.
2. B95(DE3) *ΔAΔfabR* (Addgene, catalog number: 197655). This strain is a robustly growing BL21(DE3) derivative that lacks release factor-1 (RF1), the protein responsible for translation termination at TAG codons, as well as a spontaneously mutated *fabR* gene [10]. Endogenous TAG stop codons in 95 genes were mutated to TAA or TGA to maintain cellular health and minimize unwanted readthrough due to RF1 knockout. This strain is preferred over BL21(DE3) for protein expression because by lacking RF1, TAG codon suppression by GCE machinery is more efficient, and the production of truncated protein (caused by early TAG site termination) is minimized. B95(DE3) *ΔAΔfabR* cells require lower concentrations of antibiotics to maintain normal growth rates.
3. DH10B (Thermo Fisher, catalog number: EC0113). This strain can be used for faithful propagation of plasmids and for cloning needs when users clone their genes of interest into their preferred plasmid backbone. Do not use this strain for protein expression. Though we have not explicitly tested all of them, other classical cloning strains of *E. coli* can be used in place of DH10b, including NEB 10-beta (New England BioLabs, catalog number: C3019H), DH5α (e.g., Thermo Fisher, catalog number: 18258012), NEB 5-alpha (New England BioLabs, catalog number: C2987H), or TOP10 (Thermo Fisher, catalog number: C404010).

**Plasmids**

1. pAJE3-E7 (Addgene, catalog number: 214359). Machinery plasmid for Tet2-Et incorporation that expresses a copy of the *Methanocaldococcus jannaschii (Mj)-*TyrRS-(E7)-Tet2-Et RS for faithful Tet2-Et incorporation as well as a copy of its cognate amber codon suppressing tRNA under constitutive lpp promoters. This plasmid confers spectinomycin resistance and harbors a recently developed high-copy synthetic origin of replication [1,11]. This synthetic origin of replication in pAJE plasmids can be stably propagated in cells that contain other plasmids having any of the standard or typical origins of replications, such as ColE1/pBR322/pMB1, p15A, and CDF origins, adding to the utility and versatility of this machinery plasmid.
2. pET28-sfGFP^WT^ (Addgene, catalog number: 85492). Expresses wild-type sfGFP control protein with C-terminal His6 tag, under a T7 transcriptional promoter, kanamycin resistance, and pBR322 origin of replication. sfGFP is expressed by the addition of IPTG or lactose.
3. pET28-sfGFP^150^ (Addgene, catalog number: 85493). Same as above except the sfGFP gene contains a TAG amber stop codon at site N150. The TAG codon is used to direct the translational encoding of Tet2-Et.
4. pET28-[GOI]^WT^ (you must create). Expresses your wild-type protein of interest (POI). You can clone your POI into pET28 by removing the sfGFP gene via restriction digest with NcoI and XhoI enzymes and replacing it with your gene of interest (GOI) using standard cloning techniques (e.g., ligation, Gibson Assembly, or SLiCE [12]). For the reasons mentioned above, a C-terminal purification tag is preferred if using the BL21(DE3) strain for expression. For helpful tips on construct design, see Troubleshooting tip 1.
5. pET28-[GOI]^TAG^ (you must create). Expresses your POI with Tet2-Et encoded at a TAG codon. You can clone your POI into pET28 backbone by removing the sfGFP gene by restriction digest with NcoI and XhoI and replacing it with your gene of interest (GOI) using standard cloning techniques (e.g., ligation, Gibson Assembly, or SLiCE). Using site-directed mutagenesis, change the codon to TAG (the amber stop codon) where you intend to encode Tet2-Et into your POI. See section B for recommendations on TAG site selection.

**Reagents**

**Essential reagents**

1. Tryptone (e.g., VWR, catalog number: 97063-386)
2. Yeast extract (e.g., VWR, catalog number: 97064-368)
3. NaCl (e.g., VWR, catalog number: 97061-274)
4. α-D-glucose (e.g., VWR, catalog number: 97061-168)
5. α-lactose (e.g., VWR, catalog number: AAA11074-0B)
6. Glycerol (e.g., VWR, catalog number: BDH24388.320)
7. Na_2_HPO_4_ (sodium phosphate dibasic) (e.g., VWR, catalog number: 97061-586)
8. KH_2_PO_4_ (potassium monobasic) (e.g., VWR, catalog number: BDH9268)
9. K_2_HPO_4_ (potassium dibasic) (e.g., VWR, catalog number: 97062-234)
10. NH_4_Cl (e.g., VWR, catalog number: 12125-02-9)
11. (NH_4_)_2_SO_4 _(e.g., VWR, catalog number: 7783-20-2)
12. Na_2_SO_4_ (e.g., VWR, catalog number: 7757-82-6)
13. MgSO_4 _(e.g., VWR, catalog number: 7487-88-9)
14. CaCl_2_ᐧ2H_2_O (e.g., VWR, catalog number: 10035-04-8)
15. MnCl_2_ᐧ4H_2_O (e.g., VWR, catalog number: 13446-34-9)
16. ZnSO_4_ᐧ7H_2_O (e.g., VWR, catalog number: 7446-20-0)
17. CoCl_2_ᐧ6H_2_O (e.g., VWR, catalog number: 7791-13-1)
18. CuCl_2_ (e.g., VWR, catalog number: 10125-13-0)
19. NiCl_2 _(e.g., VWR, catalog number: 7791-20-0)
20. Na_2_MoO_4_ᐧ2H_2_O (e.g., VWR, catalog number: 10102-40-6)
21. Na_2_SeO_3_ (e.g., VWR, catalog number: 10102-18-8)
22. H_3_BO_3_ (e.g., VWR, catalog number: 10043-35-3)
23. FeCl_3_ (e.g., VWR, catalog number: 7705-08-0)
24. L-arabinose (e.g., VWR, catalog number: 5328-37-0)
25. Kanamycin (e.g., VWR, catalog number: 75856-684)
26. Spectinomycin (e.g., VWR, catalog number: 89156-368)
27. Isopropyl β-D-1-thiogalactopyranoside (IPTG) (e.g., Anatrace, catalog number: I1003)
28. Agar (e.g., VWR, catalog number: 97064-336)
29. Tet2-Et amino acid (see General note 4 for reagent availability)
30. N,N-Dimethylformamide (DMF) (VWR, catalog number: BDH1117-4LG)
31. sTCO-PEG_5000_ (see General note 4 for reagent availability)
32. Sodium dodecyl sulfate (SDS) (VWR, catalog number: JT4095-4)
33. Silicone emulsion, Antifoam B^®^ (VRW, catalog number: JTB531-5)

**Reagents necessary only for defined auto-induction media**

1. Glutamic acid, Na salt (e.g., VWR, catalog number: 56-86-0)
2. Aspartic acid (e.g., VWR, catalog number: 56-84-8)
3. Lysine-HCl (e.g., VWR, catalog number: 657-27-2)
4. Arginine-HCl (e.g., VWR, catalog number: 1119-34-2)
5. Histidine-HCl-H_2_O (e.g., VWR, catalog number: 5934-29-2)
6. Alanine (e.g., VWR, catalog number: 56-41-7)
7. Proline (e.g., VWR, catalog number: 147-85-3)
8. Glycine (e.g., VWR, catalog number: 56-40-6)
9. Threonine (e.g., VWR, catalog number: 72-19-5)
10. Serine (e.g., VWR, catalog number: 56-45-1)
11. Glutamine (e.g., VWR, catalog number: 56-85-9)
12. Asparagine-H_2_O (e.g., VWR, catalog number: 5794-13-8)
13. Valine (e.g., VWR, catalog number: 72-18-4)
14. Leucine (e.g., VWR, catalog number: 61-90-5)
15. Isoleucine (e.g., VWR, catalog number: 73-32-5)
16. Phenylalanine (e.g., VWR, catalog number: 63-91-2)
17. Tryptophan (e.g., VWR, catalog number: 73-22-3)
18. Methionine (e.g., VWR, catalog number: 63-68-3)

**Solutions**

**Essential solutions**

1. LB/agar media (see Recipes)
2. 2× YT media (see Recipes)
3. SOC media (see Recipes)
4. Kanamycin stock (see Recipes)
5. Spectinomycin stock (see Recipes)
6. 1 M IPTG (see Recipes)
7. 25× M salts (see Recipes)
8. Trace metal stock solution (5,000×) (see Recipes)
9. 50× 5052 solution (see Recipes)
10. Tet2-Et solution (see Recipes)
11. ZY media (see Recipes)
12. ZY non-induction (ZY-NIM) and auto-induction media (ZY-AIM) (see Recipes)

**Solutions necessary only for defined auto-induction media**

1. Aspartate [5% (w/v), pH 7.5] (see Recipes)
2. 18 amino acid mix (25×) (see Recipes)
3. Defined-NIM and AIM media (see Recipes)

**Recipes**

1. **LB/agar media (0.5 L)**
  **Table:** 

Reagent	Final concentration	Amount
Tryptone	1 % (w/v)	5 g
Yeast extract	0.5 % (w/v)	2.5 g
NaCl	1.0 % (w/v)	5 g
Agar	1.5 % (w/v)	7.5 g
H_2_O	n/a	To 500 mL
Total	n/a	500 mL
  - After mixing reagents thoroughly, autoclave on standard liquid setting to sterilize. Note the agar will not go into solution until autoclaved.
  - After autoclaving, gently swirl the bottle to ensure molten agar is evenly mixed.
*Notes:*
*i. Store LB/agar bottle in a 55 °C oven and pour plates on an as-needed basis. LB/agar can be stored in molten form for ~2 weeks if sterility is maintained.*
*ii. If an oven is not available, plates can be poured with antibiotics once LB/agar is sufficiently cooled to touch. Plates can be stored at 4 °C for up to a week.*
2. **2× YT media (1 L)**
  **Table:** 

Reagent	Final concentration	Amount
Tryptone	1.6 % (w/v)	16 g
Yeast extract	1.0 % (w/v)	10 g
NaCl	0.5 % (w/v)	5 g
H_2_O	n/a	To 1000 mL
Total	n/a	1 L
  - After mixing reagents thoroughly, autoclave on the standard liquid setting to sterilize.
  - After autoclaving, allow it to cool to room temperature before use.
3. **SOC media (50 mL)**
  **Table:** 

Reagent	Final concentration	Amount
2× YT media	n/a	49 mL
1 M MgSO_4_	10 mM	0.5 mL
40% (w/v) α-D-glucose	0.4% (w/v) or ~20 mM	0.5 mL
Total	n/a	50 mL
  - 1 M MgSO_4_ can be made by mixing 12.3 g of MgSO_4_·7H_2_O in water up to 50 mL total volume. Adjust mass of MgSO_4_ accordingly if using a salt with a different hydration status.
  - 40% (w/v) α-D-glucose can be made by mixing 20 g of α-D-glucose with water up to 50 mL total volume. Mix thoroughly until glucose is dissolved. Gentle heating in a microwave may facilitate the dissolution of glucose.
  - Sterilize MgSO_4_, glucose, and 2× YT solutions individually by autoclaving. Allow each component to cool to room temperature and mix as indicated above. Maintain sterility while adding components together.
  - It is easy to contaminate SOC. We suggest breaking this into 5 × 10 mL aliquots before use or making smaller batches. If sterility is maintained, SOC can be stored at room temperature indefinitely. It can also be stored at -20 °C but avoid repeated freeze/thaws.
4. **Kanamycin stock (10 mL)**
  **Table:** 

Reagent	Final concentration	Amount
Kanamycin	50 mg/mL	0.5 g
H_2_O	n/a	To 10 mL
Total	n/a	10 mL
  - Sterilize by filtering with a 0.2 μm syringe-end filter.
  - Store in 1 mL aliquots at -20 °C.
5. **Spectinomycin stock (10 mL)**
  **Table:** 

Reagent	Final concentration	Amount
Spectinomycin	100 mg/mL	1 g
H_2_O	n/a	To 10 mL
Total	n/a	10 mL
  - Sterilize by filtering with a 0.2 μm syringe-end filter.
  - Store in 1 mL aliquots at -20 °C.
*Note: Do not confuse spectinomycin with streptomycin. These antibiotics are not interchangeable.*
6. **1 M IPTG (10 mL)**
  **Table:** 

Reagent	Final concentration	Amount
IPTG	1 M	2.3 g
H_2_O	n/a	To 10 mL
Total	n/a	10 mL
  - Sterilize by filtering with a 0.2 μm syringe-end filter.
  - Store in 1 mL aliquots at -20 °C.
7. **25× M salts**
  **Table:** 

Reagent	25× concentration	Amount for 25×
Na_2_HPO_4_	0.625 M	88.7 g
KH_2_PO_4_	0.625 M	85.1 g
NH_4_Cl	1.25 M	66.9 g
Na_2_SO_4_	0.125 M	17.8 g
H_2_O	n/a	to 1 L
Total		1 L
  - Add the above components to a 2 L beaker containing a magnetic stir bar. Add water up to 900 mL and mix until all components have dissolved. Add the remaining volume of water to reach 1 L.
  - Weights indicated are based on anhydrous salts. If using hydrated phosphate salts, adjust the weights accordingly to maintain indicated molarities.
8. **Trace metal stock solution (5,000×)**
  **Table:** 

Reagent	Concentration		Amount for individual 30 mL stocks
	5,000×	1×	
CaCl_2_·2H_2_O	20 mM	4 µM	8.82 g
MnCl_2_·4H_2_O	10 mM	2 µM	5.93 g
ZnSO_4_·7H_2_O	2 M	2 µM	8.62 g
CoCl_2_·6H_2_O	2 mM	0.4 µM	1.32 g
CuCl_2_	2 mM	0.4 µM	807 mg
NiCl_2_	2 mM	0.4 µM	777 mg
Na_2_SeO_3_	2 mM	0.4 µM	1.03 g
Na_2_MoO_4_·2H_2_O	2 mM	0.4 µM	1.45 g
H_3_BO_3_	2 mM	0.4 µM	371 mg
FeCl_3_	50 mM	10 µM	486 mg
H_2_O	n/a		To 30 mL
  - For each of the metals above (except FeCl_3_), make individual stock solutions using the indicated masses and dissolve in Milli-Q water up to 30 mL of total volume. Autoclave each metal solution separately to sterilize. The FeCl_3 _must be dissolved in 0.1 M HCl up to 30 mL of total volume and then filtered (through a 0.2 µm filter) to remove insoluble material and sterilize (do not autoclave).
  - Once all individual stock solutions are prepared, add 500 µL of each stock solution (except FeCl_3_) to 20.5 mL of sterile Milli-Q water. Then, add 25 mL of the FeCl_3_ solution. The total volume should be exactly 50 mL.
  - This stock solution might show minor precipitation over time but is stable at 15–25 °C for years.
9. **50× 5052 solution (500 mL)**
  **Table:** 

Reagent	Final concentration	Amount
α-D-glucose	25 mg/mL	12.5 g
α-lactose	100 mg/mL	50 g
Glycerol	25% (v/v)	125 mL
H_2_O	n/a	to 500 mL
Total	n/a	500 mL
  - Add the glucose, lactose, and glycerol components to roughly 300 mL of warm water in a 0.5 L beaker containing a magnetic stir bar. Mix until all solutions have dissolved. Additional heating may be required via microwave to encourage lactose dissolution (**CAUTION:** Remove magnetic stir bar before microwaving). Once fully dissolved, add the remaining volume of water to reach 500 mL.
  - Autoclave on liquid cycle to sterilize.
10. **Tet2-Et solution**
  **Table:** 

Reagent	Final concentration	Amount
Tet2-Et	100 mM	8.5 mg
DMF	n/a	to 275 µL
Total	n/a	275 µL
  - Vortex solution after combining to ensure all Tet2-Et has dissolved.
  - Solution can be stored at -20 °C for months but may come out of solution and require additional vortexing upon freeze/thaw cycles. For optimal expressions, prepare the solution directly before use to avoid freeze/thaw cycles.
11. **ZY media**
  **Table:** 

Reagent	Final concentration	Amount
Tryptone	1% (w/v)	10 g
Yeast extract	0.5% (w/v)	5 g
H_2_O	n/a	to 1 L
Total	n/a	1 L
  - Add the above components to a 1 L beaker containing a magnetic stir bar. Add water up to 900 mL and mix until all solutions have dissolved. Add the remaining volume of water to reach 1 L.
  - Autoclave for sterilization.
12. **ZY non-induction (ZY-NIM) and auto-induction media (ZY-AIM)**
  **Table:** 

Reagent	ZY-NIM	ZY-AIM
	**Amount**	**Amount**
ZY media	47 mL	47 mL
MgSO_4_	0.1 mL	0.1 mL
25× M salts	2 mL	2 mL
50× 5052	-	1 mL
40% (w/v) α-D-glucose	0.625 mL	-
Trace metal (5,000×)	10 µL	10 µL
Total	50 mL	50 mL
  - When preparing media, dilute the concentrated components into ZY media. Do not mix concentrated stocks and then dilute with ZY media.
  - For BL21(DE3), the final concentration for spectinomycin and kanamycin should be 100 µg/mL and 50 µg/mL, respectively. For B95(DE3) expressions, the final concentration for spectinomycin and kanamycin should be 50 µg/mL and 25 µg/mL, respectively.
  - Prepare immediately before use with a sterile technique.
13. **Aspartate [5% (w/v), pH 7.5]**
  **Table:** 

Reagent	Final concentration	Amount
Aspartate	5% (w/v)	50 g
H_2_O	n/a	To 1 L
Total	n/a	1 L
  - Mix by placing a suitable magnetic stir bar in a 2 L beaker and add 900 mL of water to the graduated cylinder. While stirring, add the appropriate amount of L-aspartic acid and adjust pH to 7.5 with 8 M NaOH. Add the remaining volume of H_2_O to bring the solution to the final volume of 1 L.
  - Sterilize by autoclaving on liquid setting.
14. **18 amino acid mix (25×) (1 L)**
  **Table:** 

Reagent	Concentration		Amount for 25×
	25×	1×	
Glutamic acid, Na salt	200 µg/mL	8 µg/mL	5 g
Aspartic acid	200 µg/mL	8 µg/mL	5 g
Lysine-HCl	200 µg/mL	8 µg/mL	5 g
Arginine-HCl	200 µg/mL	8 µg/mL	5 g
Histidine-HCl-H_2_O	200 µg/mL	8 µg/mL	5 g
Alanine	200 µg/mL	8 µg/mL	5 g
Proline	200 µg/mL	8 µg/mL	5 g
Glycine	200 µg/mL	8 µg/mL	5 g
Threonine	200 µg/mL	8 µg/mL	5 g
Serine	200 µg/mL	8 µg/mL	5 g
Glutamine	200 µg/mL	8 µg/mL	5 g
Asparagine-H_2_O	200 µg/mL	8 µg/mL	5 g
Valine	200 µg/mL	8 µg/mL	5 g
Leucine	200 µg/mL	8 µg/mL	5 g
Isoleucine	200 µg/mL	8 µg/mL	5 g
Phenylalanine	200 µg/mL	8 µg/mL	5 g
Tryptophan	200 µg/mL	8 µg/mL	5 g
Methionine	200 µg/mL	8 µg/mL	5 g
H_2_O	n/a	n/a	To 1 L
Total	n/a	n/a	1 L
  - Add 800 mL of water to a 1 L beaker, then add 5 g of each amino acid while stirring with a magnetic stir bar. Since some amino acids have trouble dissolving in solution, warming the water prior to adding the amino acids can aid in the dissolution process. It may take several hours for each component to fully dissolve. Finally, bring the volume to 1 L with water.
  - Sterilize by filtration.
  - Aliquot 45 mL of 25× 18-amino acid mix into sterile 50 mL conicals.
  - Store aliquots at -20 °C. Thaw working aliquot as needed, which can be stored stably at 4 °C for several months provided sterility is maintained.
15. **Defined-NIM and AIM media**
  **Table:** 

Reagent	NIM	AIM
	**Amount**	**Amount**
Aspartate [5% (w/v) pH 7.5]	2.5 mL	2.5 mL
50× 5052	-	1 mL
18 amino acid mix	2.0 mL	2.0 mL
25× M salts	2.0 mL	2.0 mL
MgSO_4_ (1 M)	100 µL	100 µL
Glucose [40% (w/v)]	6.25 mL	-
Trace metal solution (5,000×)	10 µL	10 µL
Sterile H_2_O	36.64 mL	41.89 mL
Total	50 mL	50 mL
  - When preparing media, add the concentrated components to sterile H_2_O, do not mix concentrated stocks, and then dilute with sterile H_2_O.
  - For BL21(DE3), the final concentration for spectinomycin and kanamycin should be 100 µg/mL and 50 µg/mL, respectively. For B95, the final concentration for spectinomycin and kanamycin should be 50 µg/mL and 25 µg/mL, respectively.
  - Prepare immediately before use with a sterile technique.

**Laboratory supplies**

1. 1.7 mL Eppendorf tubes (e.g., VWR, catalog number: 87003-294)
2. 100 mm plates (e.g., VWR, catalog number: 470210-568)
3. 500 mL graduated cylinder (e.g., VWR, catalog number: 470344-338)
4. 15 mL conical tubes (e.g., VWR, catalog number: 89126-798)
5. 50 mL conical tubes (e.g., VWR, catalog number: 89039-656)
6. 14 mL sterile culture tubes (e.g., VWR, catalog number: 60818-689)
7. 250 mL baffled flasks (e.g., VWR, catalog number: 89095-266)
8. Micro pipette tips 10 µL (e.g., VWR, catalog number: 76323-394)
9. Micro pipette tips 200 µL (e.g., VWR, catalog number: 76323-390)
10. Micro pipette tips 1,000 µL (e.g., VWR, catalog number: 76323-454)
11. 12% Mini-PROTEAN^®^ TGX^TM^ precast protein gels, 15 well, 15 µL (Bio-Rad, catalog number: 4561046)
12. Mini-PROTEAN^®^ Tetra companion running module (Bio-Rad, catalog number: 1658038)
13. Mini-PROTEAN^®^ Tetra vertical electrophoresis cell for mini precast gels, 4-gel (Bio-Rad, catalog number: 1658004)
14. Disposable PD-10 desalting column, with Sephadex G-25 resin, 1.0–2.5 mL samples (Cytiva, catalog number: 17085101)
15. TALON^®^ Superflow^TM^ (VWR, catalog number: CA71006-006)
16. Nalgene^®^ bottle-top sterile filter (Millipore Sigma, catalog number: Z358223-12EA)

## Equipment

1. Autoclave capable of sterilizing liquid media and culturing materials at 121 °C and of a saturated steam pressure of 15 PSI
2. Expression equipment:
  - Static incubator for growing LB/agar plates (set to 37 °C) (e.g., VWR, catalog number: 97025-630)
  - Shaker incubator for growing liquid cultures (e.g., New Brunswick I26R, Eppendorf, catalog number: M1324-0004)
    i. Shaker should be able to rotate at 200–250 rpm.
    ii. Refrigeration is necessary for expressions below room temperature (< 25 °C).
    iii. Shaker deck should have clamps to hold 250 mL and 2.8 L Fernbach flasks.
  - Optical density 600 nm spectrophotometer (e.g., Ultrospec 10, Biochrome, catalog number: 80-2116-30)
3. Fluorometer capable of reading sfGFP fluorescence (excitation 488 nm/emission 512 nm). Handheld fluorometers work well for routine fluorescence reads (e.g., PicoFluor from Turner Biosystems)
4. Freezer (-20 °C) for storing plasmids and antibiotics (e.g., Fisher Scientific, catalog number: 10-549-264)
5. Ice machine (e.g., Fisher Scientific, catalog number: 09-540-003)

## Software and datasets

1. ImageJ

## Procedure

**Overview**

In Part A, we discuss practical considerations for which strain to use for the expression of Tet2-protein and best strategies for preparing competent cells. In Part B, general guidelines for selecting TAG sites for your unique gene of interest are discussed. In Part C, the day-by-day steps for Tet-protein expressions at a 50 mL scale in BL21(DE3) and B95 cell lines are described in detail. Variations on autoinduction media (defined-AIM vs. ZY-AIM) and considerations when scaling up expressions are discussed where relevant. Part C also describes the evaluation of Tet2-protein reactivity after purification using a gel mobility shift assay.

- **Expression host and competent cell preparation considerations**
*Choice of expression strain*. As discussed briefly above, we recommend (and this protocol is written for) using either the BL21(DE3) or B95(DE3) *ΔAΔfabR* expression strains. Of these two, the latter RF1-deficient strain may be preferred as it limits prematurely truncated protein at TAG codons where Tet2-Et is intended to be encoded, thus increasing overall Tet2-protein yields while also allowing purification of target proteins with N-terminal purification/solubility tags without co-purification of truncated protein. Although we have not tested all other options, we expect that alternative T7-based expression strains of *E. coli* such as Rosetta(DE3), pLysS(DE3), and C41/43(DE3) are compatible with this particular Tet2-Et encoding strategy. T7Express strains from New England Biolabs (NEB) are not compatible with AIM and, therefore, methods described here will not work for these strains. We have not yet been successful at adopting Tet2-Et encoding in Origami(DE3) or Shuffle T7 strains (unpublished data).
*Tips for generating competent cells*. Expression cultures tend to be the most reproducible when multiple colonies are used to inoculate starter cultures. Thus, BL21(DE3) or B95(DE3) *ΔAΔfabR* cells need to be made sufficiently competent to transform two plasmids at once and obtain at least several dozen transformants (or colony-forming units). Note that fresh double plasmid transformations must be performed for each expression; BL21(DE3) cells (and their derivatives) should never be frozen as glycerol stocks with plasmids in them for later expressions. If cells are frozen for storage with plasmids in them, the cells will grow with the necessary antibiotics giving the false impression that they are suitable for expressions, but they will not reliably produce target protein.
  Two types of competent cells can be made: chemically competent and electrocompetent. Chemically competent cells are the less efficient option of the two, but when made properly, they are sufficiently competent to generate hundreds of colonies from a double plasmid transformation using the pAJE3-E7 and pET28-[GOI] plasmids. The advantages of chemically competent cells are that they do not require special electroporation equipment and can be prepared less frequently if users are expecting to conduct many expressions. We recommend users follow the so-called “Inoue” method when generating chemically competent cells [13]. Chemically competent cells are not recommended if triple plasmid transformations are required, as seen in Eddins et al. [1] when additional accessory plasmids were used (e.g., for expressing protein folding chaperones). In these cases, electrocompetent cells can be prepared and used with an electroporator to greatly improve the efficiency of transformation and the number of colony-forming units [14]. This protocol is written for the preferred chemical transformations. For a detailed electrocompetent cell preparation protocol, see Zhu et al. [14]. Aliquots of competent cells are stable for at least two years at -80 °C without loss of competency, provided they do not experience notable temperature fluctuations or thaw.
- **Selecting TAG sites to screen for encoding**
  When optimizing this protocol for your GOI, it is important to screen multiple TAG sites, as some protein locations are more amenable to alteration than others and some TAG sites suppress more efficiently, both of which can affect protein expression and stability. When possible, structural information of the target protein should be used to guide the placement of TAG codons so that Tet2 incorporation does not perturb protein structure or function. We find it is typically best to install Tet2 at solvent-exposed sites, within flexible loops, or residues that do not make interactions important for protein stability. Yet even with such a priori information, the ideal placement of Tet2 installation is not easily predictable, and screening multiple sites in parallel (~3–6) is often the best practice to determine the sites for efficient encoding and downstream applications without affecting protein function. For our control protein, sfGFP, we have screened for efficient encoding sites like the N150 site used in this protocol. See Figure 2 for examples of two sites that allow for high expression of sfGFP^Tet2-Et ^that follow the described guidelines.
  **Figure 2.**
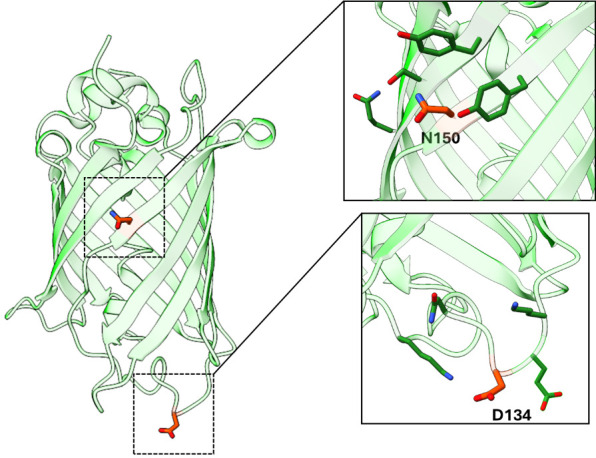
Superfolder GFP (PDB ID: 2B3P) as a model for TAG site screening. Examples of successfully encoding TAG sites N150 and D134 are highlighted. These sites adhere to TAG site placement guidelines: both sites are solvent-exposed and do not engage in structurally critical interactions, while site D134 is located within a flexible loop region.
- **Expression of Tet2-protein in BL21(DE3) and B95(DE3) cell lines**
*Note 1: Volumes of media can be changed depending on the scale of expression desired. Described below are volumes for a 50 mL sfGFP test expression.*
*Note 2: Reproducible expressions via auto-induction methods benefit from overnight non-inducing starter cultures that reach the stationary phase (total growth time ~12–18 h). AIM cultures inoculated with non-inducing cultures that did not reach the stationary phase may not always express the target protein appropriately in AIM.*
  1. Day 1: Transformations
    - Prepare two LB/agar plates, one for sfGFP^WT^ and one for sfGFP^150^ expressions, with antibiotics, as follows:
      i. Sterilize LB/agar as described above. After autoclaving, mix the contents of the bottle and allow the bottle to cool sufficiently to touch while the agar still remains liquid.
      ii. Pour 50 mL of LB/agar into a sterile 50 mL conical tube. Add 50 μL each of spectinomycin and kanamycin stock solutions for BL21(DE3) cells. For B95 cells, add 25 μL of each. Mix thoroughly and pour ~15–20 mL into each 100 mm plate. The final working concentrations of antibiotics for BL21(DE3) expressions should be 100 μg/mL spectinomycin (for the pAJE plasmid) and 50 µg/mL kanamycin (for the pET28 plasmid). The final working concentrations of antibiotics for BL21(DE3) expressions should be 50 µg/mL spectinomycin and 25 µg/mL kanamycin for BL21(DE3) or B95 cells, respectively.
      iii. Allow LB/agar to cool and solidify beside a flame with the plate lid slightly ajar for ~30 min.
      Label the plates accordingly, e.g., “pAJE3 + pET28-sfGFP^WT^” and “pAJE3 + pET28-sfGFP^150^”.
    - For each expression, label two 1.7 mL Eppendorf tubes (e.g., “pAJE3-E7 + sfGFP^WT^” and “pAJE3-E7 + sfGFP^150^”).
      Add 1 µL of pAJE plasmid (~200–400 ng) and 2 µL of pET28-sfGFP^WT^ (~100 ng) to one tube. To the other tube, add 1 µL of pAJE plasmid and 2 µL of pET28-sfGFP^150^.
    - Place both tubes containing plasmids on ice for 5 min to pre-chill.
    - Thaw aliquots of chemically competent cells [BL21(DE3) and/or B95(DE3)] and place on ice once thawed. Cells can be thawed rapidly with the warmth of your fingers, but immediately place the tube on ice once thawed.
    - Add 50 µL of competent cells to each tube with plasmids, gently mix cells with plasmids by briefly pipetting up and down or flicking gently, and place back on ice for 30 min. Do not vortex cells.
    - Heat shock cells by submerging the end of the Eppendorf tube in a 42 °C water bath for exactly 45 s.
    - Immediately place the tubes back on ice and incubate for 2 min.
    - Add 1 mL of SOC media.
*Note: Make sure SOC is not contaminated from prior use.*
    - Allow cells to recover at 37 °C with shaking at >200 rpm. It is convenient to simply tape Eppendorf tubes horizontally to the shaker deck.
      i. For BL21(DE3) cells, recover for 90 min.
      ii. For B95(DE3) cells, recover for 120 min.
    - Plate recovered culture onto LB/agar plates with appropriate antibiotics for the strain used.
      i. To ensure a sufficient number of colonies, plate all cells. To do this, centrifuge Eppendorf tubes at 3,000× *g* for 3 min, remove 900 µL of the supernatant, resuspend the cell pellet by gentle pipetting in the remaining 100 µL, then plate and spread the fully resuspended 100 µL of cells.
      ii. Let plate(s) dry with lid partially open for ~20 min near a flame (maintaining sterility) and then incubate the plate upside down overnight at 37 °C.
  2. Day 2: Non-inducing starter cultures
    Remove the LB/agar plates from the 37 °C incubator and place at room temperature or 4 °C for the day.
*Note: Several hundred colonies should be obtained for BL21(DE3) co-transformations while B95(DE3) transformation should have several dozen; see Figure 3.*
    **Figure 3.**
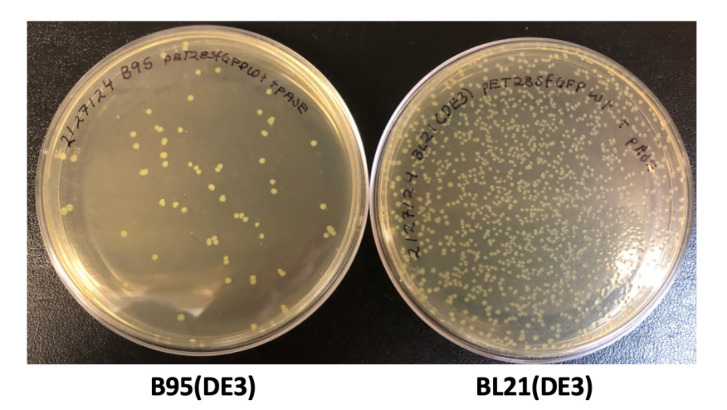
Example LB/agar transformation plates. Transformations of B95(DE3) cells (left) and BL21(DE3) cells (right) commonly produce ~100 colonies and ~1,000 colonies, respectively, after 20 h of growth at 37 °C.
    - At the end of the day (~3–5 pm), prepare starter cultures.
*Note: Defined-NIM and ZY-NIM have separate recipes as listed above. Here, researchers can choose between using defined-NIM and ZY-NIM, depending on their required expression conditions. These tetrazine GCE systems were developed and optimized using defined media because this media offers high reproducibility in expression yields and tet-encoding fidelity from batch to batch. However, the number of reagents required and the time to assemble them into defined media can be cumbersome. ZY-based media is a great alternative to defined media because it is easier to make and requires fewer reagents to assemble; however, depending on the source of tryptone and yeast extract, expression yields may vary slightly between batches. As shown in Figures 4 and 5 and Table 1, we generally see comparable Tet-protein expression yields and fidelity using ZY-based vs. defined media.*
      i. Prepare 50 mL of NIM with the appropriate antibiotic concentrations. See Recipes for details on making defined-NIM and ZY-NIM.
      ii. Prepare starter cultures for sfGFP^WT ^and sfGFP^Tet2-Et^ expressions:
      1) Label two 15 mL sterile culture tubes with the plasmid combination (i.e., pAJE3-E7 + pET28-sfGFP^WT^ and pAJE3-E7 + pET28-sfGFP^150^) and add NIM (5 mL) to each tube.
      2) To inoculate these 5 mL cultures, scrape a *glob* of cells constituting several dozen colonies from overnight LB/agar plate with a sterile pipette tip, shake the glob off into the culture media, and break apart by gentle pipetting. Enough cells should be transferred to the 5 mL starter culture such that it is slightly turbid upon inoculation.
*Note: Since expression levels can vary across different BL21(DE3) clones, we recommend inoculating starter cultures with several dozen colonies to obtain an averaged population for highly reproducible results from one expression to another.*
      3) Grow starter cultures at 37 °C with shaking at 250 rpm overnight.
      **Figure 4.**
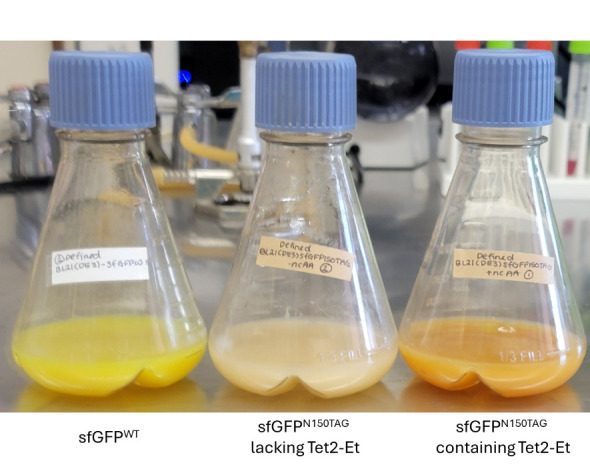
Example BL21(DE3) defined-AIM expressions displaying the characteristic green color of sfGFP^WT^ (left) and orange color of sfGFP^Tet2-Et ^(right). Cultures expressing pET28-sfGFP^150^ and lacking Tet2-Et amino acid should be colorless since no full-length sfGFP is expressed (middle).
      **Figure 5.**
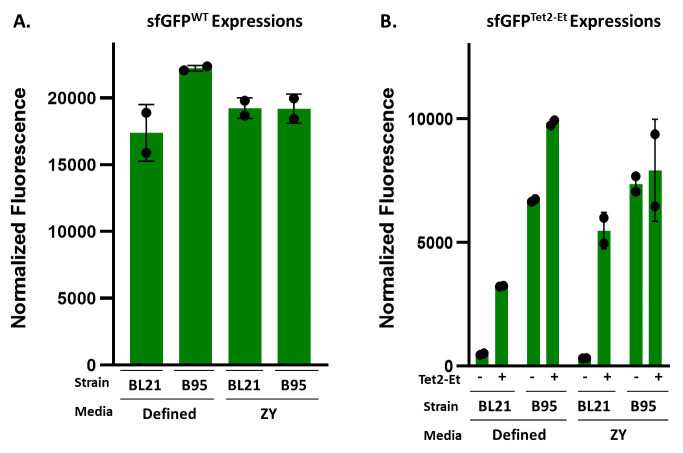
Expected sfGFP fluorescence values of 50 mL expression cultures for (A) sfGFP^WT ^and (B) sfGFP^Tet2-Et^. By plotting the normalized fluorescence of each expression (i.e., the raw fluorescence values divided by the OD_600_), relative yields per cell can be estimated for each expression. It is important to remember that oxidized encoded Tet2-Et quenches sfGFP’s fluorescence and so these values do not necessarily reflect the actual yield of sfGFP^Tet2-Et^ protein. sfGFP^Tet2-Et^ normalized fluorescence values shown in this representative expression should give approximate fluorescence values to expect for control sfGFP expressions. B95(DE3) expressions lacking Tet2-Et ncAA display expected high normalized fluorescence values due to near-cognate suppression [1]. These expressions were performed in duplicate and allowed to express for 20 h.
      **Table 1.** Characteristic yields (milligrams per liter of culture) for sfGFP^WT^ and sfGFP^Tet2-Et^ from purified 50 mL expressions using 500 mL of TALON affinity resin calculated using absorbance values at A_280_

Media condition	Cell line	sfGFP variant	Yield (mg/L per 500 μL TALON resin)
**Defined-AIM**	BL21(DE3)	WT	250
		sfGFP^Tet2-Et^	230
	B95(DE3) *ΔAΔfabR*	sfGFP^WT^	210
		sfGFP^Tet2-Et^	160
**ZY-AIM**	BL21(DE3)	sfGFP^WT^	240
		sfGFP^Tet2-Et^	230
	B95(DE3) *ΔAΔfabR*	sfGFP^WT^	170
		sfGFP^Tet2-Et^	160
  3. Day 3: Expressions
*Note 1: Expression cultures should have a starting OD of ~0.05 after inoculation.*
*Note 2: A 50 mL expression is best grown in a 250 mL baffled flask. It is important to use baffled flasks as they increase the aeration of expressions, promoting proper growth of* E. coli *and higher protein expression levels. Increased aeration, in turn, increases the formation of foam, making it necessary to use antifoam in expression cultures.*
*Note 3: When scaling for larger expressions, consider the size of the baffled flask in reference to the volume of media used. We recommend a total flask volume that is ~3–5 times the volume of the media to ensure adequate aeration, e.g., a 2 L flask works well with 500 mL of media.*
*Note 4: We recommend mixing all media stock components together in a single batch and then dividing working media into appropriate 250 mL baffled culture flasks to ensure all expressions contain the same media. Below, we perform 3* × *50 mL expressions: one for sfGFP^WT^, one for sfGFP^150^ with Tet2-Et in the media, and one for sfGFP^150^ lacking Tet2-Et in the media. In this case, prepare 150 mL of AIM and split into three flasks.*
    - Preparing and inoculating auto-induction expressions
      i. Measure the OD_600_ of starting cultures after overnight growth. OD_600 _readings are a measurement of culture density through light scattering and transmittance of a given culture—optical density at a 600 nm wavelength. This measurement provides an easy assessment of the *E. coli* culture’s growth phase, allows inoculation of expression cultures at equal densities, and provides normalization of protein production by cell density. OD_600 _for NIM is generally low and is expected to be between 1.5 and 4, depending on the culture vessel, media type used, and metabolic burden of constitutively expressed protein components.
      ii. Dissolve Tet2-Et ncAA in DMF as described in Recipes. We recommend making the stock with ~10% more volume than is needed for expressions. For example, for a 50 mL expression, you need 250 µL of 100 mM Tet2-Et solution to reach 0.5 mM final concentration, and so we recommend making ~275 µL of stock for this expression.
      iii. Prepare defined-AIM or ZY-AIM as described in Recipes.
      After adding all reagents and antibiotics, and before adding Tet2-Et, split the media into *Tet2-Et-containing* and *Tet2-Et-lacking* expression batches.
      1) For Tet2-Et-containing expressions, add 250 μL of 100 mM Tet2-Et stock to each 50 mL expression (0.5 mM Tet2-Et final).
      2) For expressions lacking Tet2-Et (wild-type expressions and minus ncAA culture expressions), add 250 μL of DMF (100%) for each 50 mL expression.
      iv. Inoculate expression cultures with non-inducing overnight cultures so that the starting OD_600_ upon incubation is 0.05 (e.g., if overnight starter cultures have an OD_600 _of 5, add 0.5 mL to a 50 mL culture)
      v. Add antifoam to each culture to enable proper aeration. Two drops or ~50 µL should be sufficient to eliminate foam in 50 mL cultures.
*Note: For 1 L cultures, add 6 drops or ~150 μL.*
      vi. Grow at 37 °C at 250 rpm for 20–24 h.
*Note: If expressing a protein that requires lower expression temperatures, monitor OD_600_ until it reaches ~1.5, then lower to the desired temperature, and continue culturing for another 16–24 h.*
  4. Day 4: Evaluating expressions and harvesting cells
*Note 1: After expression, it is convenient to estimate the amount of sfGFP produced by measuring culture fluorescence, since only full-length sfGFP (and not protein that was prematurely truncated at the TAG codon) will fluoresce. Yields determined by fluorescence are not directly comparable between sfGFP^WT^ and sfGFP^Tet2-Et^ as the Tet moiety affects fluorescence properties (see Figure 1, and General note 2 for information on tetrazine redox and quenching).*
    - After 20–24 h of expression, measure OD_600_ and fluorescence of sfGFP^WT^ and both sfGFP^150^ expressions (Tet2-Et-containing and Tet2-Et-lacking cultures; ex/em: 485/510 nm). The sfGFP^WT^ culture should be visibly green, while the sfGFP^150^ expression culture should be orange (quenched sfGFP, Figure 4). The OD_600_ values will vary depending on the target protein. Normal values will range from ~2.5 to 15. Final OD_600_ values below 2 are indicative of poor cell growth and/or protein expression. Characteristic fluorescence values for all discussed expression conditions are summarized in Figure 5.
    - Harvest cells by centrifugation.
      i. Centrifuge cells at 5000–10,000× *g* for 10–20 min at 4 °C and then decant or aspirate the media.
      ii. Resuspend the cell pellet in the appropriate buffer for the downstream application, and either store at -80 °C (flash-freezing cells in liquid nitrogen may help maintain the integrity of unstable proteins) or proceed with purification.
      1) Buffer choice is often contingent on protein purification strategy.
      2) Here, the buffer can be supplemented with a cryoprotectant [e.g., 10% (v/v) glycerol] to minimize adverse effects associated with freezing sensitive or unstable proteins.
      3) For His_6_-tagged proteins to be purified via TALON resin, a recommended resuspension/lysis buffer would be 50 mM Tris pH 7.5, 500 mM NaCl, 10% (v/v) glycerol, and 5 mM imidazole. Avoid the use of reducing agents such as DTT or b-mercaptoethanol as they can react with (reduce) and temporarily inactivate the Tet2 amino acid [1].
- **Evaluation of Tet2-protein reactivity with sTCO-PEG_5000_ and other sTCO-probes**
**Purifying Tet2 proteins: important considerations**
  - The sfGFP^WT^ and sfGFP^Tet2^ proteins expressed above contain C-terminal His6 tags and can be purified using TALON of Ni-NTA metal affinity resins according to manufacturers’ recommendations.
  - Avoid the use of reducing agents during the purification of Tet2 proteins as they can reduce the Tet2 amino acid, rendering it temporarily unreactive.
**Quantifying purified Tet2-containing proteins: important considerations**
  - Quantifying Tet2-protein concentration can be performed with normal standard methods including Bradford, BCA, Lowry assay, or measuring protein absorbance at 280 nm (A_280_) using UV-VIS as described below. See General note 2 for more information on accurate Tet2-protein concentration determination using A_280_ measurements.
  - For sfGFP^WT^ and sfGFP^Tet2-Et^ proteins, the molar extinction coefficients at 280 nm (ε_280_) of 24,080 and 37,640 M^-1^·cm^-1^ can be used, respectively [1].
    - The concentration can be determined using Beer’s Law: A_280_ = ε_280_ × l × c where l is the pathlength (usually 1 cm) and c is the concentration of protein.
    - Example: An A_280_ of 1.8 with a 1 cm path length corresponds to 76 μM sfGFP^WT^ or 48 μM sfGFP^Tet2-Et^.
**Evaluating Tet2-protein reactivity with sTCO-PEG_5000_**
  Accurate Tet2 encoding and its reactivity on a protein of interest is most easily assessed by measuring electrophoretic mobility shifts upon conjugation with sTCO-PEG_5000_. After successful conjugation, the attached PEG polymer will slow the migration of reactive target protein while unreactive protein will migrate identical to that of wild-type (unmodified) protein. This assay requires only a few minutes for the reaction to occur and about 1 h to run a standard SDS-PAGE gel, and only small quantities (<10 μg) of protein are needed. While treatment of Tet2-protein with other sTCO reagents can be used to ensure the Tet2-protein is reactive (e.g., by visualizing in-gel fluorescence after reacting with an sTCO-fluorophore), it is not trivial to evaluate the extent or the stoichiometry of conjugation with these methods. See General note 1 for preparation and handling of sTCO-PEG_5000_ and General note 2 regarding tetrazine reactivity. As a quick and clear diagnostic test, in the following assay, a 10-fold molar excess of sTCO-PEG_5000_ is reacted with purified Tet2-protein for ~5 min, and then excess sTCO-PEG_5000_ is *quenched* with free Tet2 amino acid to eliminate potential nonspecific reaction with the protein prior to boiling for SDS-PAGE analysis.
  1. Prepare a 1 mM stock of sTCO-PEG_5000_ stock in water and a 20 mM Tet2-ethyl stock in DMF. Very little Tet2-Et is needed for each quenching reaction; we recommend making 100 µL of Tet2-Et at 100 mM in DMF as described in Recipes and then diluting this to 20 mM. The stock can be frozen at -20 °C and repeatedly thawed for quenching steps.
  2. For each 30 μL reaction, 10 μM sfGFP^WT^ or sfGFP^Tet2-Et^ protein is incubated with or without 100 μM sTCO-PEG_5000_ and then quenched with ~1 mM Tet2-Et amino acid. Table 2 shows an example reaction scheme in which the sfGFP^WT^ and sfGFP^Tet2-Et^ protein concentrations were determined to be 50 and 20 μM by A_280_ measurement.
    **Table 2.** sTCO-PEG_5000 _reactions

	sfGFP^WT^	sfGFP^WT^ + sTCO-PEG_5000_	sfGFP^Tet2-Et^	sfGFP^Tet2-Et^ + sTCO-PEG_5000_
Buffer (50 mM Tris pH 7.5, 100 mM NaCl)	24 μL	21 μL	15 μL	12 μL
Protein (10 μM final)	6.0 μL	6.0 μL	15 μL	15 μL
1 mM sTCO-PEG_5000 _ (100 μM final)	0 μL	3 μL	0 μL	3 μL
Final volume	30 μL	30 μL	30 μL	30 μL
	*Incubate 5 min at room temperature*			
20 mM Tet2 amino acid (~1 mM final)	2 μL	2 μL	2 μL	2 μL
	*Incubate 5 min at room temperature*			
4× Laemmli loading buffer [*containing + 10% (v/v) β-mercaptoethanol*]	10 μL	10 μL	10 μL	10 μL
	*Denature by incubation at 95 °C for 5 min*			
	*Centrifuge samples for 2 min at >10,000× g*
  3. Run 10–15 μL of each sample on a 12% SDS-PAGE gel. This equates to running ~2 μg of each sample.
  4. Stain the SDS-PAGE gel using common Coomassie staining methods and evaluate the reactivity of Tet2-protein with sTCO-PEG_5000_ (Figure 6). This can be done qualitatively, by estimating the amount of protein that has shifted, or quantitatively, by taking a high-resolution scan of the gel and using densitometry software (e.g., ImageJ) to evaluate the exact percentage of reacted sTCO-PEG.
    **Figure 6.**
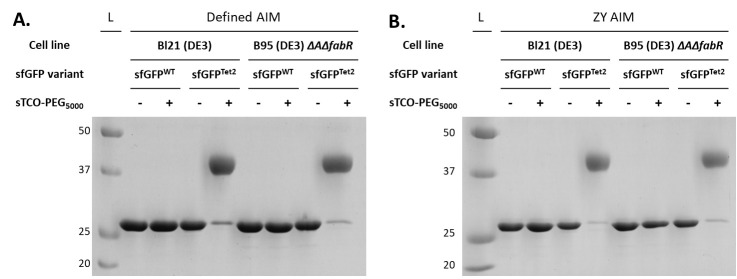
sTCO-PEG_5000_ gel mobility shift assay to evaluate the reactivity of purified Tet2-protein expressions. Proteins expressed in defined-AIM (panel A) and ZY-AIM (panel B) media were evaluated for their reactivity by conjugating with a 10-fold molar excess of sTCO-PEG_5000_. A clear upward electrophoretic shift due to the added molecular weight of PEG_5000_ is observed for sfGFP^Tet2-Et ^proteins but not sfGFP^WT^, confirming the specificity of Tet2-Et labeling. Complete (> 95%) reactivity is observed for proteins expressed in either BL21(DE3) or B95(DE3) *ΔAΔfabR* cell lines. See General notes 1–3 for considerations when Tet/TCO reactivity is not complete.

## Validation of protocol

This protocol or parts of it has been used and validated in the following research article:

- Eddins et al. [1]. Truncation-Free Genetic Code Expansion with Tetrazine Amino Acids for Quantitative Protein Ligations. Journal name (Figure 4, panel D) (Figure S9).
- Two biological replicates were used to produce the dataset in the protocol and the dataset referenced.

## General notes and troubleshooting

**General notes**

1. Considerations for Tet-TCO labeling reactions:
  - *TCO structure and reactivity*: TCO molecules can come in a variety of forms, having different stabilities and rates of reactivity with tetrazines. In this protocol, we describe the use of strained *trans*-cyclooctene (sTCO) functionalized reagents (containing the fused cyclo-propyl ring, Figure 1) for conjugation to encoded tetrazines because of their ultra-fast reactivity with tetrazines. sTCO displays second-order kinetics approximately two orders of magnitude higher than standard TCO [15], which greatly reduces conjugation time and the requirement for excess TCO reagent to ensure complete reactivity. Currently, sTCO reagents are not commercially available; however, methods to synthesize them have been well-established [5,15]. To accelerate access to sTCO reagents, we offer a select assortment of sTCO reagents (as well as tetrazine reagents, see General note 4) at the GCE4All Center ( https://gce4all.oregonstate.edu/ , gce4all-center@oregonstate.edu). TCO reagents that lack the strain-promoting cyclopropyl group of sTCO are commercially available and are effective for conjugating tetrazine-containing proteins. If using TCO-functionalized reagents, incubation times will need to be increased ~10-fold to compensate for slower reaction times; however, additional optimizations will be necessary depending on TCO used [15].
  - *TCO stability*: sTCO and TCO may undergo spontaneous *trans*-to-*cis* isomerization over time. Users should be mindful of how they store these reagents to maximize stability. We have found that large TCO-PEG polymers are relatively stable at -20 for months and appear to be stable dissolved in water at high concentrations. We recommend minimizing the number of freeze/thaw cycles of TCO stocks by aliquoting samples when they are received. TCO-PEG polymers can lose reactivity over time so when attempting to use stoichiometric amounts of label, it may be worth determining the percent reactivity of a TCO-label stock by titrating the sample with freshly prepared Tet-protein.
  - *Causes of non-reactive Tet2-Et protein.* Testing reactivity with the sTCO-PEG mobility shift assay validates successful tetrazine encoding when reactions are successful; however, if a reaction does not occur, tetrazine may still be encoded but is in a reduced (unreactive) state (see General note 2 below), the encoding site is buried and inaccessible to labeling reagents, the TCO reagents have degraded, or natural amino acids were encoded at the TAG/UAG codon instead of Tet2-Et. Natural amino acid encoding at amber codons is typically caused by a deficiency in the GCE machinery such that insufficient amounts of amber-suppressing tRNA amino-acylated with Tet2 are generated, allowing endogenous tRNAs to wobble-base pair their anticodons with the UAG codon so that natural amino acids are encoded instead. The most common natural amino acid encoded at UAG codons via near-cognate suppression is glutamine, and such events are more common in RF-1 deficient expression strains [e.g., B95(DE3)]. However, as shown and discussed in Eddins et al. [1], the described Tet2-Et GCE system utilized here was optimized specifically for its ability to routinely and effectively out-compete any near-cognate suppression events in B95(DE3) or BL21(DE3) cells. Mass spectrometry (see General note 3 below) can help delineate whether any non-reactive protein was caused by near-cognate suppression, e.g., if the MS spectra of sfGFP^Tet2-Et ^protein contain a peak with a mass consistent with glutamine at site N150 instead of Tet2-Et; then, the GCE machinery system was not functioning adequately and improvements in expression parameters must be considered or more carefully followed as described.
2. Tetrazine reduction—considerations for reactivity and protein quantification:
  The tetrazine amino acid can undergo reversible reduction when exposed to reducing environments. Reduction of free Tet2-Et amino acid by the cells in the culturing media may be observed during protein expression, as indicated by a media color change from pink to clear. Reduction of Tet2-Et amino acid during protein expression can be minimized using baffled flasks that provide high rates of aeration, high shaking rates (220–250 rpm), and antifoam to maximize air exchange rates. Once encoded into a protein, the reducing environment of the *E. coli* cytoplasm may cause the Tet2 residue to be reduced, but it will oxidize and be fully reactive to sTCO once purified and buffer exchanged (Figure 1). Some considerations on tetrazine redox properties are provided below.
  - *Promoting the oxidized state of encoded Tet2 for complete bioconjugation*: After cell lysis, exposure to ambient oxygen during purification and desalting/buffer exchange will cause the encoded, reduced Tet2-Et to spontaneously oxidize to the reactive form (see Eddins et al. [1]). This oxidation event occurs rapidly after purification and desalting, only requiring a few minutes (up to 1 h at most) for nearly all encoded Tet residues on sfGFP^Tet2-Et^ to oxidize. The kinetics of oxidation may change depending on the target protein and site of encoding. If proteins are stable overnight at 4 °C, allowing them to oxidize in the fridge after purification and after buffer exchanging into a buffer of choice lacking imidazole (extended exposure to imidazole at high concentrations can inhibit tetrazine reactivity), with a closed cap, is typically sufficient to achieve complete oxidation and maximal reactivity. If proteins are unstable, perform a reactivity-over-time assessment to determine when a given Tet2-protein will be fully oxidized. If overnight incubation is not sufficient for complete oxidation, an additional desalting/buffer exchange step may help.
  - *Effect of Tet2-Et redox state on sfGFP fluorescence.* The N150 site in sfGFP resides in close physical proximity to its chromophore, and so tetrazine encoded at this site will quench sfGFP fluorescence when the tetrazine is in its oxidized state, but not when in its reduced form (see Figure 1 [16]). Consequently, when the encoded Tet2-Et is oxidized, sfGFP^Tet2-Et^ is orange in color and is reactive to sTCO, but when it is reduced, the sfGFP^Tet2-Et^ will be fluorescent green and will not be reactive to sTCO. After a successful reaction with sTCO, the oxidized sfGFP^Tet2-Et^ will change from orange color to fluorescent green (see Video S1). This restoration of sfGFP^Tet2-Et^ fluorescence upon exposure to sTCO is a convenient strategy to evaluate successful reaction and labeling.
  - *In-cell fluorescence of sfGFP^Tet2-Et^*: Because the redox state of tetrazine influences its ability to quench sfGFP fluorescence, and because it is difficult to quantify the ratio of oxidized to reduced sfGFP^Tet2-Et^ when inside the cell, it can be difficult to measure how much sfGFP^Tet2-Et^ is produced after expressions using in-cell fluorescence. Still, the fluorescence values plotted in Figure 5 are representative sfGFP^Tet2-Et^ values for a 20-h expression at this scale. As an approximate rule of thumb, when tetrazine is fully oxidized, sfGFP^Tet2-Et^ fluoresces at approximately one-sixth the amount of sfGFP^WT^.
  - *Estimating Tet2 protein concentration using A_280_*: Tet2-protein concentration can be determined using standard methods including UV light absorption, Bradford, BCA, or Lowry assays. It is convenient to estimate Tet-protein concentrations using UV-VIS and Beer’s Law; however, using an accurate molar extinction coefficient is necessary. Encoded Tet2-Et affects the extinction coefficient significantly when the residues tetrazine group is in its oxidized form, but to a much lesser extent when in reduced form. For consistency of protein quantification, we emphasize the importance of providing sufficient incubation time for all Tet2-protein to oxidize. To calculate the approximate extinction coefficient (ε_280_) of a Tet2-containing protein, use the following formula:
    ε_280_ = (# of Tet2 residues) × (13,560 M^-1^·cm^-1^) + (# of Trp residues) × (5,500 M^-1^·cm^-1^) + (# of Tyr residues) × (1,490 M^-1^·cm^-1^)
3. Mass spectrometry to evaluate encoding fidelity:
  - Mass spectrometry enables direct evaluation of tetrazine ncAA encoding fidelity and can help delineate sources of non-reactive protein. We recommend using whole-protein mass spectrometry to evaluate tetrazine encoding fidelity in new proteins and expression constructs. We also recommend comparing tetrazine-containing protein to the wild-type variant so that the expected differences in mass can be confirmed (see Figure 7) Note that it is possible that no off-target MS peaks are detected even though notable quantities of non-reactive protein are observed in the sTCO-PEG_5000_ gel mobility assays. In these cases, the lack of reactivity is likely caused by the presence of multiple independent non-reactive protein species that individually are in too little abundance to be detected by MS but collectively are observed as a single aggregate band in the electrophoresis assays. Still, this appears to be a negligible amount of unreactive protein.
    **Figure 7.**
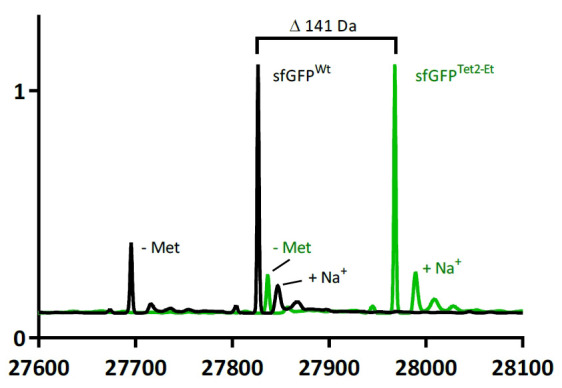
ESI-mass spectrometry comparison of sfGFPTet2-Et (green) and sfGFPWT (black). sfGFPWT: expected mass 27,827 Da, observed 27,826.5 Da. sfGFPTet2-Et: expected mass 27,968 Da, observed 27,967.8 Da. Observed mass difference is 141.3 Da, matching the expected 141 Da difference. Commonly observed methionine loss and sodium adduct peaks are highlighted. This data was adapted from Eddins et al. [1].
  - Site-specificity of Tet2-Et encoding can be confirmed by tryptic digestion of target proteins followed by MS/MS sequencing methods. Since different peptide fragments ionize with different efficiencies, these fragmentation methods should only be used to confirm the site of encoding and should not be used to assess the fidelity of encoding.
4. Availability of tetrazine and TCO reagents:
  - Tet2-Et
    i. Tet2-Et is available for purchase from the GCE4All Center product #1001 (see https://gce4all.oregonstate.edu/tetrazine-amino-acids).
    ii. See Blizzard et al. [17] for synthesis protocol.
  - TCO reagents
    i. sTCO-PEG_5000_ can be requested via email at gce4all-center@oregonstate.edu.
    ii. See Bednar et al. [5] or Fang et al. [15] for synthesis protocol.
    iii. TCO-coupled reagents are available from various commercial vendors.

**Troubleshooting**

1. Improving protein expression efficiency
  While the sfGFP^Tet2^ protein should express efficiently if following the above protocol, optimization may be necessary to achieve efficient expression of biologically relevant proteins containing the Tet2 amino acid.
*Construct design:*
  - Placement of affinity purification tag, N-terminus vs. C-terminus: C-terminal affinity purification tags are preferred when expressing Tet2 proteins in BL21(DE3) cells to avoid the co-purification of truncation products. If N-terminal tags are preferred, or if the target protein is not stable or compatible with non-native C-terminal affinity tags, ion-exchange and size-exclusion chromatography can be used to separate some full-length proteins from their truncation products. If TAG codons are placed in close proximity to the N-terminus, then truncation products may be sufficiently small that they get degraded or are insoluble, and therefore they will not be co-purified with N-terminal affinity tags. Alternatively, proteins can be expressed in B95(DE3) *ΔAΔfabR* cells and no truncation product will be produced.
  - Solubilization fusion proteins: fusing target proteins with N-terminal solubilizing fusion proteins such as maltose binding protein (MBP), glutathione-S-transferase (GST), and small ubiquitin modifying protein (SUMO) can often increase expression yields and solubility of challenging target proteins. Adding a proteolytic cleavage sequence (e.g., TEV, 3C) between the fusion protein and the target protein allows users to remove the solubility fusion protein during purification processes, so they do not interfere with downstream assays. SUMO proteins can be cleaved with their cognate SUMO proteases. See Esposito and Chatterjee [18] for additional information on using fusion proteins as solubility tags.
2. Alternative expression strategies
*Manual induction*: We generally find auto-induction expression methods as described above the most efficient strategy for encoding Tet2 into proteins. However, manual induction methods in which an inductant (e.g., IPTG when using pET vectors) is added to the media to initiate protein expression are also compatible with the pAJE-E7 expression system [1]. For example, protein can be expressed by growing freshly transformed BL21(DE3) cells in a standard rich media (e.g., LB, 2× YT) at 37 °C until cultures reach mid-log phase (e.g., OD_600_ ~0.6–0.8) and adding IPTG to a final concentration of 0.5–1 mM and Tet2-Et to 0.5 mM. Expression temperature can be adjusted to be between 18 and 37 °C as needed, and protein can be expressed for 3–24 h. As with standard manual induction and expression of wild-type proteins, it is important to optimize conditions (e.g., inductant concentration, temperature, media). These factors should be optimized using wild-type target protein constructs and TAG-interrupted proteins in parallel, including sfGFP controls.
3. Best practices for using ImageJ/other densitometry software to evaluate reactivity
  - Quantification of protein reactivity can be useful for determining the success of tetrazine fidelity, redox, and the stability of TCO probes. It is important to use densitometry software properly, without biasing the extent of reactivity in either direction. Because PEG mobility shifts cause a spreading effect on SDS page gels (see diffuse of higher MW bands in Figure 6), the best practice is to measure the remaining area of the band of unreacted protein instead of the area of the shifted protein.
  - When possible, it is best to use area boxes of equal sizes for each band. SDS-PAGE gels can sometimes have variable sizes for lanes, which may require multiple sizes of area boxes to be used.
  - Reactivity quantification is less accurate for gels that have a high background signal or gels with overloaded protein lanes. Background subtraction tools can be used to account for this; however, destaining SDS gels properly will help eliminate these concerns.

## Supplementary information

The following supporting information can be downloaded here:

1. Video S1. sfGFP^Tet2-Et ^Reaction with sTCO-PEG_5000 _
